# Post-transcriptional Regulation of Gene Expression in Plants during Abiotic Stress

**DOI:** 10.3390/ijms10073168

**Published:** 2009-07-10

**Authors:** Maïna Floris, Hany Mahgoub, Elodie Lanet, Christophe Robaglia, Benoît Menand

**Affiliations:** 1 Aix-Marseille Université, Laboratoire de Génétique et Biophysique des Plantes, Marseille, F-13009, France; E-Mails: maina.floris@univmed.fr (M.F.); hany.mahgoub@etumel.univmed.fr (H.M.); elodie.lanet@univmed.fr (E.L.); christophe.robaglia@univmed.fr (C.R.); 2 CNRS, UMR Biol Veget & Microbiol Environ, Marseille, F-13009, France; 3 CEA, DSV, IBEB, Marseille, F-13009, France

**Keywords:** plants, abiotic stress, gene expression, post-transcriptional regulations

## Abstract

Land plants are anchored in one place for most of their life cycle and therefore must constantly adapt their growth and metabolism to abiotic stresses such as light intensity, temperature and the availability of water and essential minerals. Thus, plants’ subsistence depends on their ability to regulate rapidly gene expression in order to adapt their physiology to their environment. Recent studies indicate that post-transcriptional regulations of gene expression play an important role in how plants respond to abiotic stresses. We will review the different mechanisms of post-transcriptional regulation of nuclear genes expression including messenger RNA (mRNA) processing, stability, localization and protein translation, and discuss their relative importance for plant adaptation to abiotic stress.

## 1. Introduction

**Figure 1.**
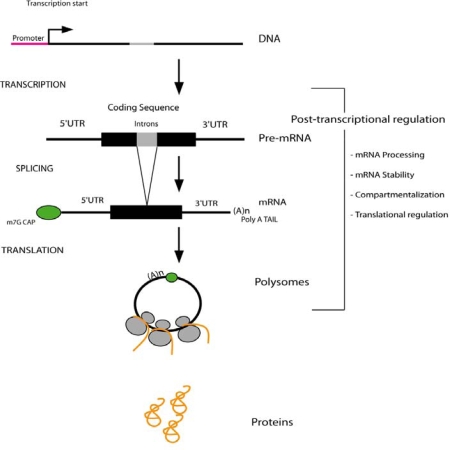
Post-transcriptional regulations of gene expression. Gene expression should be controlled both at transcriptional and post-transcriptional level in order to fine-tune protein production. We define post-transcriptional regulations by all the regulated steps from mRNA to protein synthesis (mRNA processing, stability, compartmentalization translation).

Land plants are anchored in one place for most of their life cycle and therefore must adapt their physiology and development to environment variables such as light intensity, temperature and the availability of water and nutrients. For example, plants always have to deal with sunlight intensity, which varies along the day and also at the seasonal scale. On one hand, excess of light leads to cellular damage due to oxidative stress triggered by reactive oxygen species (ROS) accumulation. On the other hand, low light intensity results in a reduced growth of the plant due to diminished photosynthesis. Another example of the big impact of environment on plant growth is the availability of nutrients (such as phosphate and nitrate) and water, which are often limiting in the soil. Thus, subsistence of plants depends on the rapid regulation of gene expression in order to adapt their physiology to abiotic stresses. The expression of nuclear genes is highly regulated at both transcriptional and post-transcriptional level. Post-transcriptional regulations of gene expression occur at the levels of pre-messenger RNA (mRNA) processing (capping, splicing, and polyadenylation), mRNA stability, and mRNA translation (Figure 1). Here we will review the evidences of these mechanisms of post-transcriptional regulation during abiotic stress response in plants.

## 2. Regulation of mRNA Processing

Transcription of protein-encoding genes gives rise to precursor mRNAs that are capped, polyadenylated and spliced before being translated into proteins. Splicing results in the excision of introns sequences from the pre-mRNA and is mediated by the spliceosome. Alternative splicing allows production of more than one mRNA from a single gene. Bioinformatics and experimental data indicate that 30% of the *Arabidopsis thaliana* (thale cress) transcripts may be alternatively spliced [1,2].

SERINE/ARGININE-RICH (SR) proteins are a part of the spliceosome and act as splicing regulators in eukaryotes. In *A. thaliana*, cold and heat stresses regulate the alternative splicing of the pre-mRNAs for many SR encoding genes with different splicing functions under stress conditions [3]. Using a genetic screen, Lee *et al.* identified *STABILIZED1 (STA1)*, a gene coding for a nuclear pre-mRNA splicing factor that is important under cold stress conditions in *A. thaliana* [4]. The *Sta1-1* mutant shows a defect in the splicing of the *COLD-REGULATED 15A* (*COR15A*) pre-mRNA leading to hypersensitivity to chilling, to salt stress and to the stress responsive hormone abscissic acid (ABA). Expression of *STA1* itself is up-regulated under cold stress. These results show that STA1 is involved in regulating the splicing and turnover of transcripts and allows resistance to cold stress.

Several lines of evidence indicate that RNA-BINDING PROTEINS (RBPs) also have a crucial role in the regulation of mRNA splicing. RBPs are characterized by the presence of an RNA Binding Domain made of an RNA recognition motif. GLYCINE-RICH-RNA BINDING PROTEINS (GR-RBPs) have an N-terminal RNA recognition motif and a C-terminal tail of variable length enriched in glycine residues [5]. Plant GR-RBPs have been implicated in responses to changing environmental conditions, particularly cold stress [6,7]. Plants over-expressing *GR-RBP* show a better tolerance to cold stress. GR-RBPs might regulate the processing and/or the stability of mRNAs that are highly expressed during stress conditions. They can act as chaperones that modulate RNA-RNA interactions or shuttle the mRNA for efficient processing. Under stress conditions, GR-RBPs may stabilize mRNA either during the transfer from the nucleus to cytoplasm or directly in the cytoplasm, allowing efficient mRNA processing.

OLIGO URIDYLATE BINDING PROTEIN 1 (UBP1) is another protein implicated in mRNA stability and pre-mRNA splicing. UBP-ASSOCIATED (UBA) proteins are *A. thaliana* nuclear RBPs that interact with UBP1 [8]. Transient expression of *UBA1* in protoplasts leads to an increased accumulation of mature reporter mRNA, independently of splicing efficiency [9]. This data suggests that UBA proteins may stabilize mRNA in the cytoplasm. ABA-ACTIVATED PROTEIN KINASE (AAPK) - INTERACTING PROTEIN 1 (AKIP1) from *Vicia faba* (bean) is a homolog of *A. thaliana* UBA1. AKIP1 interacts with the guard cell protein AAPK that controls stomatal pores aperture and ions channels [10,11]. AAPK phosphorylates AKIP1 after treatment with ABA. Once phosphorylated, AKIP1 binds and stabilizes mRNA encoding the DEHYDRIN protein, which is implicated in cell protection. Together these data suggest that splicing is a step at which post-transcriptional regulation occurs during stress.

Another level of regulation through mRNA processing was revealed by a forward genetic approach. Zhang *et al.* identified *oxt6* (*oxidative stress tolerant 6*), an *A. thaliana* mutant that tolerates oxidative stress [12]. The *oxt6* mutation was caused by a T-DNA insertion in *At1g30460,* a gene encoding the *A. thaliana* ortholog of the 30-kD subunit of the CLEAVAGE AND POLYADENYLATION SPECIFICITY FACTOR CPSF30. Wild-type growth and stress susceptibility of *oxt6* could be restored by expression of *CPSF30*. The authors suggest that the CPSF30 protein is involved in the processing of pre-mRNA prior to polyadenylation [13]. Therefore, a deficit of CPSF30 might alter the site of polyadenylation for particular mRNAs. Zhang *et al*. showed that the poly(A) site choice is different between *oxt6* and the wild-type plants and is dependent on the presence of CPSF30 [12]. Together, these results indicate that a polyadenylation factor subunit can influence stress tolerance responses. All these data reveal the importance of mRNA processing regulation for stress tolerance in plants.

## 3. Regulation of mRNA Stability by RNA Silencing

RNA silencing is a mechanism involved in gene expression regulation during plant development, responses to virus infection and in response to abiotic stress [14]. While this mechanism has been first described in transgenic plants, it was further shown to be conserved among eukaryotes [15]. RNA silencing implicates short RNA molecules that inhibit gene expression in a sequence specific manner at the level of transcription, mRNA stability or translation. Endogenous small RNAs (sRNAs) from 20 to 25 nt are processed from non-coding double-stranded (ds) RNA precursors by RNAses of the DICER-LIKE (DCL) family [15]. One strand of the sRNAs duplex is then loaded into an ARGONAUTE (AGO) protein to form the so-called RISC (RNA Induced Silencing Complex) complex. This ribonucleoproteic complex recognizes mRNAs that present a partially or fully complementary sequence to the sRNA. Once sRNA and mRNA hybridize, the RISC complex silences expression of the target mRNA by triggering its cleavage and/or inhibition of its translation [16,17]. There are various classes of sRNAs, mainly differing by their biogenesis. Most known post-transcriptional regulations mediated by RNA silencing involve microRNAs (miRNAs). miRNAs are produced from *MIRNA* precursors, called pri-miR, that form an intramolecular double strand hairpin structure[18].

### 3.1. Implication of sRNAs in Abiotic Stress

The first indication that RNA silencing could be involved in abiotic stress responses was provided by the computational identification of plant sRNAs and their corresponding mRNA targets [19]. Several of these potential targets encode stress response proteins such as SUPEROXIDE DISMUTASES, LACCASES and ATP SULFURYLASES (APS). At the same time, sRNAs differentially regulated or specifically expressed under stress were cloned from *A. thaliana* seedlings grown under stress conditions [20]. More recently, a microarray-based large-scale analysis identified fourteen stress-regulated miRNAs that were induced by high salinity, drought or cold [21]. A computational approach in *A. thaliana*, confirmed by transcriptome experiments, has also shown that eight miRNAs are differentially expressed in response to low temperature [22]. Further analysis of the *MIR* loci encoding these miRNAs revealed known stress-related *cis*-regulatory elements in their promoter regions [22]. These data suggest that RNA silencing is an important component of the plant stress response pathway.

Few sRNAs have been characterized in detail for their involvement in abiotic stresses such as oxidative, nutrient and salt stress. Among these few examples, we can distinguish two mechanisms: (1) sRNAs that are induced under stress conditions and which repress negative regulators of stress tolerance, and (2) sRNAs whose expression is downregulated by stress to allow the accumulation of positive regulators of stress tolerance. Below we present three examples that illustrate the action of these two mechanisms in response to abiotic stress conditions.

### 3.2. sRNAs Up-Regulated by Stress

#### 3.2.1. miR399 and Phosphate Starvation

**Figure 2.**
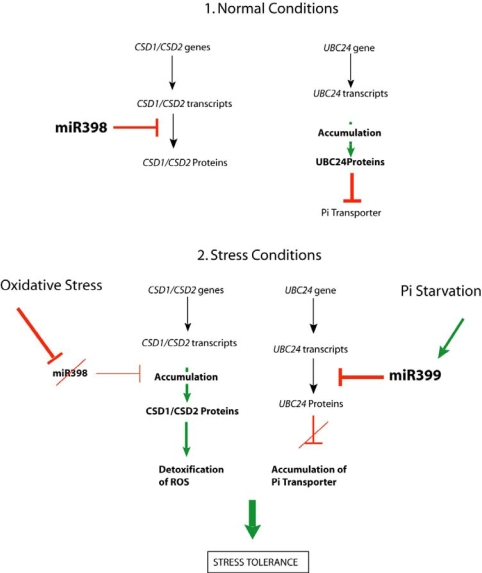
Stress conditions can induce opposite regulations of miRNAs accumulation. The two miRNAs miR398 and miR399 are implicated in post-transcriptional regulation of gene expression under stress conditions. They are oppositely regulated. miR398, which targets *COPPER SUPER OXIDE DISMUTASES* (*CSD1* and *CSD2*), is constitutively expressed in normal conditions and repressed under oxidative stress. [31]. By contrast, miR399 is produced only under phosphate starvation in order to repress the negative regulator of Pi import *UBC24* (*UBIQUITIN CONJUGATING ENZYME 24*) [25–27].

Phosphorus (P) is an essential nutrient for plant growth and development. Therefore plants have developed a set of responses to enhance inorganic phosphate (Pi) uptake under phosphate starvation. These responses involve increasing Pi uptake activity, modification of root architecture, secretion of organic acids or phosphatase, remobilization of internal P and association with mycorrhizial fungi [23]. Among the large number of genes involved in the phosphate metabolism pathway, *UBC24* (*At2g33770*) encodes UBIQUITIN CONJUGATING ENZYME 24, which is involved in the targeted protein degradation pathway [24]. UBC24 regulates Pi transporters availability to prevent nutrient overloading. Bioinformatics analysis identified six loci in *A. thaliana* and eleven in *Oryza sativa* (rice) that might be targeted by a miRNA called miR399 [19]. This miRNA has five potential target sites in the 5’-untranslated region (5’UTR) of the *UBC24* transcript and two sites appear predominant for miR399-guided cleavage. Pi deficiency induces reduction of the *UBC24* transcript amount in parallel to an induced transcription of the *MIR399* precursor [25,26]. Accumulation of miR399 appears to be a specific response to Pi deficiency, as it does not occur under any other stresses tested [26] [27]. Transgenic plants overexpressing *MIR399* do not accumulate *UBC24* mRNA under high Pi, confirming that miR399 negatively regulates *UBC24* expression [26]. Furthermore miR399 overexpression results in the over-accumulation of Pi in the shoot and the appearance of the symptoms of Pi toxicity. These experiments suggest that the regulation of *UBC24* by miR399 plays an important role in the Pi signalling pathway and is part of the adaptive response to Pi starvation (Figure 2).

#### 3.2.2. Natural Antisense sRNAs and Salt Stress

Another class of sRNAs, called natural antisense small interfering RNAs (nat-siRNAs), has been identified from plants exposed to salt stress [28]. Nat-siRNAs are produced from intermolecular dsRNA formed by complementary transcripts. In the example described by Borsani *et al.* the *P5CDH* (*Δ-PYRROLINE-5-CARBOXYLATE DESHYDROGENASE*) gene, that encodes a proline catabolism enzyme, is constitutively transcribed under normal conditions [28]. Another gene, *SRO5* (*SIMILAR TO RADICAL-INDUCED CELL DEATH ONE 5*), is transcribed from the same DNA locus as *P5CDH* but on the opposite direction. Whereas only *P5CDH* is transcribed under normal conditions, salt stress activates transcription of both genes, leading to the accumulation of two partially complementary mRNAs. The resulting dsRNA is cleaved by DCL in order to produce nat-siRNAs duplexes. These siRNAs are incorporated into the RISC complex and trigger silencing of *P5CDH* and *SRO5*. This partial inhibition of proline catabolism allows a better tolerance to salt stress. Bioinformatics analyses predict the existence of more than two thousand pairs of natural antisense transcripts in *A. thaliana* [29,30]. It can be expected that nat-siRNAs represent a common way for environmental stress response in plants and possibly in other eukaryotes.

### 3.3. sRNAs Down Regulated by Stress (miR398)

Stress conditions such as drought, cold, salinity, high light or metal toxicity result in the accumulation of ROS in plant cells [32]. To prevent excess cellular damage, reactive radicals are scavenged at the site of their synthesis in the chloroplast. SUPEROXIDE DISMUTASES (SOD) are the firsts scavengers for the detoxification of superoxide radical O_2_^−^ allowing conversion of O_2_^−^ into H_2_O_2_^−^ [32]. SODs are expressed under stress conditions to enable detoxification and are repressed under normal conditions. In *A. thaliana*, *CSD1* and *CSD2* mRNA, that encode Cu/Zn-SOD (CSD) proteins, accumulate in response to treatments inducing oxidative stress, such as high light or excess Cu^2+^and Fe^3+^. However, nuclear run-on assays indicate that *CSD1* and *CSD2* are equally transcribed under normal or oxidative conditions, indicating that their mRNAs are post-transcriptionally regulated [31]. Indeed, under normal growth conditions, the two *CSD* genes are transcribed but their mRNAs are silenced through the action of miR398. miR398 is present in *A. thaliana* and *O. sativa* and could be encoded from three loci [31]. In response to oxidative stress, the locus *MIR398* is transcriptionally down regulated, resulting in the absence of miR398 (Figure 2). Reduction of miR398 abundance allows the accumulation of *CSD1* and *CSD2* mRNAs, which can therefore be translated. Transgenic plants carrying a miR398-resistant mutation in the *CSD2* mRNA show better tolerance to oxidative stresses than wild type plants. This suggests that the fine regulation of *CSDs* transcript stability by miR398 is important to rapidly reduce ROS production in response to oxidative stress. Thus, in *A. thaliana*, miRNA mediated down-regulation of positive regulators of stress tolerance appears to be an important mechanism controlling oxidative stress responses.

## 4. General Evidences of Translational Regulations in Plants

**Figure 3.**
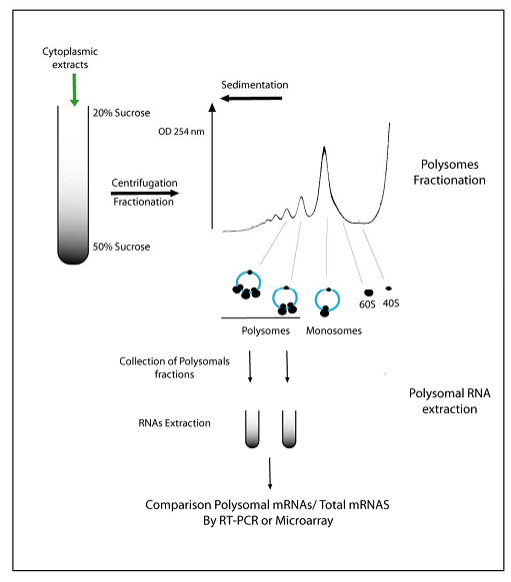
Polysomes fractionation on sucrose gradients allows the isolation of actively translated mRNA. Cytoplasmic extracts are separated on a sucrose gradient. After centrifugation, fractions are collected from the bottom to the top of the gradient under continuous reading of optical density at 254 nm. The major peak corresponds to monosomes (entire 80S ribosome assembled on mRNA). Lower in the gradient are polysomes (translationally active mRNAs associated with more than one ribosome). Free RNAs and other low molecular weigh components of the cytoplasm sediment at the top of the gradient. RNAs can be extracted from the different fractions and quantified by RT-PCR or microarray to determine which mRNAs are translated. Comparing accumulation of mRNA in polysomal RNA *vs*. total RNA allows the identification of potential translational regulations.

In all organisms, regulation of mRNA translation allows fine modulation of the level of protein synthesized from its corresponding mRNA. Translation efficiency of individual mRNA can be estimated through the evaluation of the amount of mRNA associated with translating ribosomes [33]. mRNAs that recruit multiple ribosomes are actively translated and form ribonucleotidic complexes known as polysomes. Fractionation of polysomes on a sucrose gradient is an ancient method that has been adapted for plant material [34] (Figure 3). Differential mRNA translation occurs in response to numerous environmental stimuli such as heat stress [35], salt stress [36], water deficit [37], oxygen deprivation, pathogen infection [38]**,** and sucrose starvation [39]. In one these studies**,** leaves of *A. thaliana* have been placed under normal or dehydration conditions [40]. The proportion of individual mRNA in polysomes has been measured for over two thousand genes. The authors reported that the majority of mRNAs show a significant decrease in polysomes association in response to dehydration stress. While some transcripts are upregulated under stress, their association with polysomes is maintained at the same level as in normal conditions. These results suggest that the effect of dehydration on translational level varied between mRNA species.

In a similar microarray experiment, the differential translation of 25,607 *A. thaliana* transcripts was analysed in response to sucrose starvation in cell culture [39]. This study identified 224 mRNAs that were regulated translationally and 268 mRNAs that were regulated transcriptionally. Most of the translationally regulated mRNAs are repressed by sucrose starvation, which is consistent with a general decrease of metabolic activity. The authors also observed that transcripts related to the protein synthesis machinery and to cell cycle control were particularly abundant among the translationally regulated transcripts. These results suggest that translational control may be important for gene regulation in response to sugar starvation.

A more recent study has shown that hypoxia stress followed by reoxygenation, which is linked with cellular ATP content, promoted adjustment in the level of polysomes in *A. thaliana* seedlings [41]. These data suggest that translational regulations contribute to the adaptation of plants to environmental perturbation by limiting consumption of ATP and directing the synthesis of specific proteins. Therefore differential translation of mRNAs appears to be a key component in the response to oxygen deprivation and reoxygenation.

Overall, we can conclude from the data mentioned above that many mRNAs undergo translational changes during environmental adaptation. Although the amount of polysomes bound mRNAs has been rarely correlated with the amount of the corresponding protein, polysomes analysis appears to give an acceptable estimation of translational efficiency.

## 5. Involvement of Untranslated Regions (UTRs) in Translational Regulations

**Figure 4.**
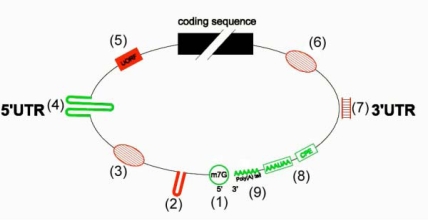
Generic structure of a eukaryotic mRNA, illustrating some of the post-transcriptional regulatory elements that affect gene expression. Numbers on the 5’UTR refer to: (1) m7G, 7-methyl-guanosine cap; (2) hairpin-like secondary structures; (3) interacting repressor protein; (4) internal ribosome entry sites (IRES); (5) upstream open reading frame (uORF). Numbers on the 3’UTR refer to: (6) repressor protein complex; (7) sRNAs binding sites; (8) cytoplasmic polyadenylation elements (CPE) and hexanucleotide AAUAAA polyadenylation signal; (9) poly (A) tail. Red-coloured elements usually down-regulate translation of the main coding sequence, whereas green-coloured elements are usually favourable for translation of the main coding sequence.

**Figure 5.**
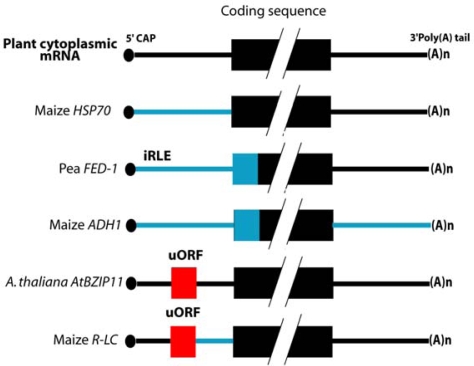
Cis-acting elements involved in translation of plant cytoplasmic mRNAs. The 5’ 7-methyl-guanosine cap structure is indicated by a filled circle. Regions of mRNAs with cis-acting sequences that regulate translation are indicated by blue boxes. Upstream open reading frames (uORFs) are indicated by red boxes. *HSP70*, *HEAT SHOCK PROTEIN 70* [45]; *FED-1*, *FERREDOXIN*-1; iLRE, internal light-regulatory element [47]; *ADH1, ALCOHOL DEHYDROGENASE-1* [43]; *AtBZIP11, A. thaliana BASIC LEUCINE ZIPPER 11* [48].

mRNA features that contribute to translational regulation under abiotic stress have been studied using a comparison of the abundance of the total mRNAs versus polysomal mRNAs in leaves of *A. thaliana* [42]. It was shown that translation of an mRNA is mostly affected by control elements located within untranslated regions (5’UTR and 3’UTR). In addition, nucleotides surrounding the initiation codon also influence translational efficiency (figure 4). The interactions between 5’ and 3’ UTR might promote a synergistic enhancement of mRNA translation, ensuring that scanning proceeds from the correct end. Electroporation of mRNAs constructs into protoplasts was used to investigate involvement of maize *ADH1* (*ALCOHOL DEHYDROGENASE-1*) mRNA features in translation efficiency. The authors found that the 5’UTR, a portion of the coding sequence and the 3’UTR of *ADH1* mRNA are all required for its efficient expression in hypoxic protoplasts [43]. A more recent analysis of *ADH1* expression indicates that an internal ribosome entry site (IRES) in the 5’UTR is implicated in translational regulations [44]. In heat-shocked protoplasts, mRNA electroporation studies have also shown that the 5’-UTR of *HSP70* (*HEAT-SHOCK PROTEIN 70*) mRNA is sufficient for translational enhancement under stress [45] (Figure 5). Another study investigated the role of 5’UTR on translation of *FERREDOXIN-1* (*FED-1*) mRNA. Light can induce an increased association of *FED-1* mRNA with polysomes which is mediated by an internal light-regulatory element (iLRE) located in the 5’UTR and in the coding sequence [46]. In addition they proposed that *FED-1* mRNA stability is correlated with its association with polysomes [47]. Overall, all these data indicate that the features of the 5’UTR and the 3’UTR of mRNAs are involved in their translation in response to environmental stresses.

### 5.1. Upstream Open Reading Frames (uORFs) in Plants

Some of the most important translational control signals in eukaryotes are upstream open reading frames (uORFs) that are located in the 5’UTR [49]. The earliest example of uORF-guided translational regulation under stress was that of *GCN4* mRNA in yeast [50]. The presence of uORFs in 5’UTR modulates translation efficiency of the main ORF due to preferential recruitment of ribosomes. Factors such as uORF length, the nucleotide context of the start and stop codons, and the sequence between the uORF and the main ORF, all contribute to affect the efficiency of translation of the main ORF [51]. Depending of these characteristics of the uORF, ribosomes may reach the main ORF through reinitiation or leaky-scanning [52,53].

Five uORFs have been characterized in monocots, including two examples in rice that are uORFs of *S-ADENOSYLMETHIONINE DECARBOXYLASE* (*ADOMETDC*) gene [54] and *MYB7* (*MYB DOMAIN PROTEIN 7*) gene [55], and two examples in maize: *OPAQUE-2* gene [56], *R-Lc* gene [29]. Also, uORFs have been found in *A. thaliana* genes such as *AtBZIP11* (*A. thaliana BASIC LEUCINE ZIPPER 11*) [48], and *ARF* (*AUXIN RESPONSIVE FACTOR*) genes [57]. A recent study has used *O. sativa* and *A. thaliana* full-length cDNAs sequences to determine the prevalence of uORFs [58]. These bioinformatics analyses predict the presence of uORFs in the 5’UTR of a subset of genes conserved between both species. Many of these genes encode proteins that have a regulatory function including transcription factors, signal transduction factors, developmental signal proteins, a homolog of the translation initiation factor eIF5 (eukaryotic TRNASLATION INITIATION FACTOR 5), and a RING finger protein. Another recent study has identified several genes whose translation may be regulated by uORFs in monocotyledons [59]. The uORFs identified in these studies are strong candidates to trigger translational control.

The data mentioned above suggest that uORFs are important cis-acting regulatory elements present in the 5’UTR of many plants mRNAs. Several examples illustrate the role of uORFs in translational regulation during development, but only a few examples illustrate their role in response to abiotic stress. We will now discuss examples of uORFs that repress the translation of mRNAs under abiotic stress conditions in plants.

### 5.2. Examples of Translational Regulation Mediated by uORF

S-ADENOSYLMETHIONINE DECARBOXYLASE (SAMDC) is a key enzyme of the polyamine biosynthesis pathway [60]. Two overlapping small uORFs consisting of 3 and 52 codons respectively are present in the 5’UTR of *SAMDC* genes. The 52 codons uORF is responsible for the translational repression of *SAMDC* gene under normal growth conditions [61]. Another study demonstrated that both uORFs are involved in translation repression in response to stress conditions and negative feedback controlled by polyamines [62]. This is an example illustrating the metabolite-dependent translational control involving conserved uORFs in plants.

**Figure 6.**
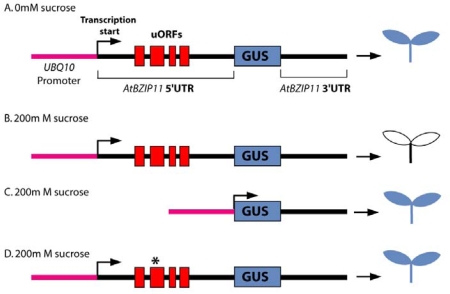
Effect of the *AtBZIP11* (*A. thaliana BASIC LEUCINE ZIPPER 11*) 5’UTR on sucrose-induced repression of translation in *A. thaliana* seedlings. Schematic illustration of an *AtBZIP11* 5’UTR controlled β*-GLUCURONIDASE* (*GUS*) chimera terminated by the *AtBZIP11* 3’UTR and fused to the *POLYUBIQUITIN10* (*UBQ10*) promoter (A and B). Histochemical staining of 5 days-old seedlings grown without sucrose (A) shows GUS expression in the root and shoot, while seedlings grown with 200 mM sucrose (B) show repression of GUS expression via a mechanism called sucrose-induced repression of translation. Deletion of the *AtBZIP11* 5’UTR destroys sucrose-induced repression of translation as indicated by presence of GUS expression in the root and shoot (C). A single mutation (*) in uORF2 is sufficient to destroy sucrose-induced repression of translation (D). Adapted from [48].

Another study reported that the expression of the gene coding for the AtBZIP11 transcription factor is post-transcriptionally regulated by sucrose [63]. High sucrose concentrations result in translational repression of the *AtBZIP11* expression. The 5’UTR of *AtBZIP11* mRNA is necessary for the sucrose-induced repression of translation (SIRT) (figure 6). The long *AtBZIP11* 5’-UTR (547nt) contains four uORFs called uORF1, uORF2, uORF3 and uORF4 coding for polypeptides of respectively 18, 42, 5 and 18 amino acids. The upstream ORF2 is highly conserved in 5’UTRs of other *A. thaliana* BZIP genes as well as in other dicotyledons and monocotyledons plants [48]. The involvement of *AtBZIP11* uORFs in sucrose-induced repression of translation (SIRT) has been investigated via the introduction of point mutations in single uORFs in transgenic plants. These plants carry the *A. thaliana POLYUBIQUITIN10* (*UBQ10*) promoter and the *AtbZIP11* 5’UTR fused to the reporter gene coding for β-GLUCURONIDASE (GUS) (Figure 6) [48,64]. The exchange of the internal putative start codon of uORF2 to a stop codon leads to GUS activity when plants are grown in high sucrose concentration (Figure 6D). This indicates that SIRT activity is dependent on translation of the C-terminal part of uORF2. Thus, the authors have proposed the name “Sucrose Control uORF” (SC-uORF) for the uORF2 of *AtBZIP11* and any orthologous uORF from other plants or other *A. thaliana* genes. A recent study investigated in detail the mechanism of translational regulation of *AtBZIP11* by SIRT [65]. These authors show that the SC-peptide is required and sufficient for SIRT. They also suggest that the SC-peptide interacts with other molecules to repress translation of the main ORF. Overall, these results suggest that a sucrose-sensing pathway controls translation of several plants *BZIP* mRNA harbouring the conserved uORF in their 5’UTRs. This illustrates an example of a metabolite-dependent translational control system involving a conserved uORF in plants.

## 6. Compartmentalization of mRNAs in the Cytoplasm

During the recent years, a new aspect of post-transcriptional regulation of gene expression has been uncovered: the sequestration of mRNAs in the cytoplasm. Using tomato cell cultures, Nover *et al.* provided the first evidence of electron dense cytoplasmic foci, assembled under heat stress, called Heat Stress Granules (HSG) [66]. A recent study using tobacco and *A. thaliana* shows that HSG can be distinguished from stress granules (SG) that are not dependent on heat shock proteins (HSP) [67]. Following stress exposure, a subset of mRNAs aggregate with specific proteins, allowing physical separation of these mRNAs from the translational machinery and resulting in transient translational repression. Specific RNA binding proteins and the eukaryotic TRANSLATION INITIATION FACTOR (eiF4E) were identified as markers of stress granules in plants and in animals [68]. Understanding mechanisms of mRNA sorting by localization of mRNAs in distinct structures within the cytoplasm, and the resulting reversible regulation of translation, will be an important challenge for future studies on post-transcriptional regulation [68,69].

## 7. Conclusions

The work reviewed here indicates that, under abiotic stress, plant genes are regulated at all post-transcriptional stages, from mRNA processing to protein translation. Similar stresses can induce different mechanisms of post-transcriptional regulation, indicating that there is no preference for one regulatory mechanism for a particular stress.

mRNA stability seems to be an important mode of regulation both during and after mRNA processing. However, the precise function of RNA binding proteins during mRNA processing is not yet very well understood and needs more investigation. Responses to abiotic stress highlight fascinating examples of the different modes of action by which sRNAs can regulate mRNA stability. On the one hand, a sRNA induced by stresses represses negative regulators of stress tolerance and, on the other hand, repression of the expression of a sRNA by stress allows the accumulation of positive regulators of stress tolerance. The role of sRNA might also have been overlooked given that they can regulate translation as well as RNA stability [16,17]. New examples of sRNAs-mediated regulation of translation in response to stress might be reported soon.

Most of the examples of translational regulation in response to stress involve the 5’UTR and particularly uORFs. However, there may be a bias because uORFs are particularly easy to identify and study compared to other elements in the 5’ and 3’UTRs, such as secondary structure or sites for recognition by regulatory RNA binding proteins.

We anticipate that future studies will uncover that more plant genes are post-transcriptionally regulated in response to abiotic stress and that several mechanisms are operating together. For example, during the root nodule development induced by symbiotic bacteria in the legume plant *Medicago*, *truncatula* the spatial and temporal expression of transcription factor MtHAP2-1 (*M. truncatula* HEME ACTIVATOR PROTEIN homolog 2-1) is regulated by both a miRNA and a uORF [70,71]. This highlights the importance of investigating the combinatory, possibly synergistic, roles that multiple regulatory mechanisms may play in the regulation of plant gene expression during environmental variations.

Many papers mentioned in this review argue that post-transcriptional regulations of gene expression may be particularly important during stress responses because it allows more rapid adaptation in the proteome than transcriptional regulation can provide. Therefore, post-transcriptional regulations seem to be important for rapid adaptation of gene expression in response to environmental variations in plants.
